# Highly sensitive microdisk laser sensor for refractive index sensing via periodic meta-hole patterning

**DOI:** 10.1515/nanoph-2024-0598

**Published:** 2025-01-30

**Authors:** Haerin Jeong, Nu-Ri Park, Byoung Jun Park, Moohyuk Kim, Jin Tae Kim, Myung-Ki Kim

**Affiliations:** KU-KIST Graduate School of Converging Science and Technology, 34973 Korea University, Seoul 02841, Republic of Korea; Quantum Technology Research Department, Electronics and Telecommunications Research Institute (ETRI), Daejeon 34129, Republic of Korea; Center for Quantum Information, Korea Institute of Science and Technology (KIST), Seoul 02792, Republic of Korea

**Keywords:** microdisk laser sensor, meta-hole patterning, metasurface, whispering gallery mode (WGM), on-chip optical sensor

## Abstract

Microdisk lasers have emerged as compact on-chip optical sensors due to their small size, simple structure, and efficient lasing capabilities. However, conventional microdisk laser sensors face challenges in enhancing interactions with external analytes, as their energy remains predominantly confined within the laser material. In this study, we present a novel microdisk laser sensor incorporating periodic meta-hole patterning, designed to enhance external interaction while maintaining the integrity of the whispering gallery mode (WGM). Numerical simulations show that in an InGaAsP microdisk laser (5 μm diameter, 250 nm thickness), the WGM remains stable with periodic meta-holes (period *a* = 340 nm, diameter *d* < 0.4*a*), achieving a resonant wavelength near 1,500 nm. The inclusion of meta-holes led to a substantial improvement in sensitivity, reaching up to 100.8 nm/RIU – a 2.26-fold increase over nonpatterned microdisks. Experimental validation confirmed lasing in structures with a *d*/*a* ratio of 0.32, achieving a maximum sensitivity of 74.5 nm/RIU, which represents a 2.02-fold enhancement compared to nonpatterned designs. This advancement in microdisk laser design not only opens new possibilities for high-performance, miniaturized optical sensors but also holds significant potential for integration into next-generation on-chip sensing technologies.

## 1 Introduction

With the advancement of modern technology, the demand for compact and efficient on-chip optical sensor technology has been increasing rapidly [1], [2], [3]. These miniaturized sensors play a critical role in various fields, such as biological sensing [4], [5], [6], [7], [8], environmental monitoring [9], [10], [11], [12], and chemical detection [13], [14], [15], [16], [17], where high sensitivity and compactness are essential. Traditionally, passive on-chip sensors using evanescent coupling with passive on-chip resonators have been widely used for these applications [18], [19], [20]. However, their performance is often limited by sensitivity to environmental fluctuations and the requirement for precise and stable coupling with the resonators [21], [22], which restricts their effectiveness in real-world environments. To overcome these challenges, active laser-based sensors have emerged as a more reliable and efficient alternative [23], [24], [25]. Unlike passive sensors, active sensors benefit from the stability of stimulated emission, which maintains a consistent resonant linewidth, independent of environmental conditions [26], [27], [28], [29]. This intrinsic stability enables active sensors to operate effectively in less controlled environments, enhancing their versatility. Additionally, with separated pump and signal wavelengths, active sensors can simplify measurement setups and reduce the need for complex and expensive equipment. These advantages have positioned active laser sensors as promising candidates for next-generation optical sensing technologies.

Among the various active laser-based sensor designs, microdisk lasers have gained considerable attention due to their structural simplicity and ease of laser driving, making them particularly suitable for practical applications [30], [31]. While other cavities with extremely small mode volumes, such as photonic crystal slot cavities [32] and photonic crystal edge cavities [33], demonstrate exceptional performance owing to their enhanced light–matter interaction and high sensitivity to refractive index changes, microdisk resonators remain of significant interest across various fields due to their unique advantages, including straightforward fabrication, scalability, and seamless integration with on-chip systems. Microdisk lasers leverage whispering gallery mode (WGM) resonance, where light circulates along the disk’s edge, facilitating highly confined resonant modes. However, in conventional microdisk lasers, a significant portion of the resonant energy remains confined within the laser cavity, limiting interactions with external substances. This confinement reduces the overall sensitivity of the sensor, posing challenges in detecting low concentrations of analytes or subtle environmental changes, thus restricting their effectiveness in critical applications.

Efforts to enhance the sensitivity of microdisk laser sensors have largely focused on deforming the geometric structure of the disk to increase interaction with external analytes [34], [35], [36], [37]. While these modifications have shown potential, they often compromise key performance aspects, such as the symmetry of the resonant modes, ultimately degrading the laser’s overall functionality.

In this study, we address these limitations by introducing a novel approach: incorporating periodic meta-holes into the microdisk laser structure. These subwavelength meta-holes are designed to enhance external interactions without compromising the original profile of the WGM. Our numerical simulations reveal that by introducing periodic meta-holes into an InGaAsP microdisk laser (5 μm in diameter, 250 nm thick, with a hole periodicity *a* of 340 nm and hole diameters *d* less than 0.4*a*), we can preserve the WGM near the wavelength (*λ*) of 1,500 nm while significantly improving sensitivity. Specifically, the sensitivity increased to 100.8 nm/RIU – representing a 2.26-fold enhancement compared to nonpatterned structures. Experimental evaluations further confirmed the lasing performance of the patterned microdisk laser, achieving a sensitivity of 74.5 nm/RIU with a *d*/*a* ratio of 0.32, marking a 2.02-fold improvement. These results underscore the potential of meta-hole patterned microdisk lasers as a transformative technology for high-performance, next-generation optical sensing applications.

## 2 Meta-hole patterned microdisk laser sensor

**Figure 1:**
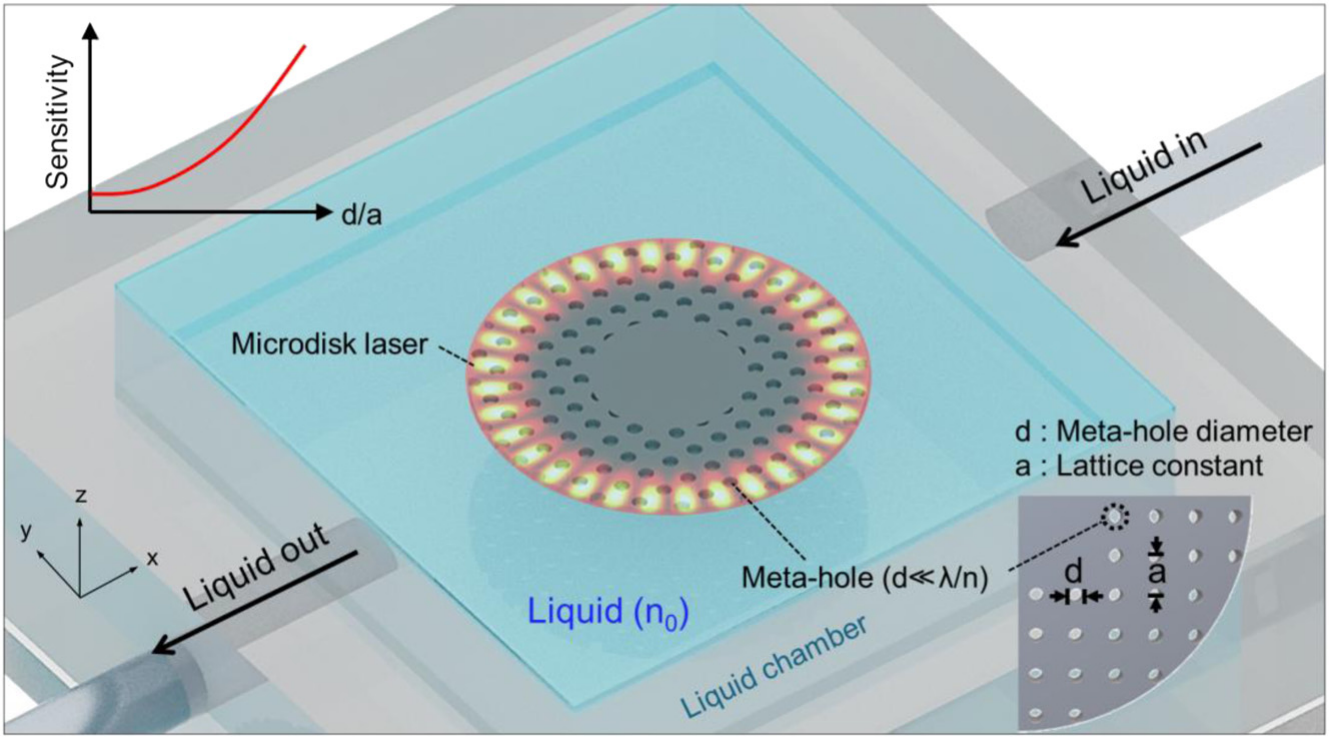
Schematic of meta-hole patterned microdisk laser sensor and its operating principle. The microdisk laser features subwavelength meta-holes arranged in a periodic lattice to enhance interaction with the surrounding liquid medium, significantly enhancing sensor sensitivity while preserving the whispering gallery mode profile. Constructed from InGaAsP (*n* = 3.45) with a diameter of 5 μm and a thickness of 250 nm, the microdisk is optimized to operate near a wavelength of 1,500 nm. The lattice constant of the meta-holes is denoted by *a*, and the hole diameter by *d*. A liquid chamber enables controlled flow of liquid with a refractive index of *n*_0_ around the sensor, with changes in *n*_0_ causing measurable shifts in the laser’s resonant wavelength.

Figure 1 illustrates the structural design and operational principle of the proposed microdisk laser sensor with periodic meta-hole patterning. The design integrates subwavelength meta-holes (*d* ≪ *λ*/*n*, where *n* is the refractive index of the resonator) arranged in a periodic lattice within the microdisk resonator [38]. Among the various hole patterns, we chose a periodic hole pattern to allow for the application of diverse processing techniques in creating meta-holes. This meta-hole arrangement lowers the effective refractive index of the WGM by enhancing its interaction with external analytes, while preserving the WGM profile. This significantly increases the sensor’s sensitivity without disrupting the WGM.

The microdisk laser, composed of InGaAsP (*n* = 3.45), is designed to operate at a wavelength near 1,500 nm. The microdisk has a diameter of 5 μm and a thickness of 250 nm. The meta-hole periodicity *a* is fixed at 340 nm, while the hole diameter *d* is varied to investigate the sensitivity variation of the laser sensor. For liquid sensing, a small chamber is integrated with the InGaAsP microdisk laser, allowing liquid (with refractive index *n*_0_) to flow around the sensor. Variations in the liquid’s refractive index cause shifts in the laser’s resonant wavelength, enabling the detection of subtle changes in the liquid’s optical properties. The incorporation of a meta-hole pattern amplifies the interaction between the external liquid, resulting in a significantly enhanced shift in the resonant wavelength (see Supplementary Information). This study intentionally employs a straightforward design approach using a rectangular lattice of meta-holes, focusing on simplicity and scalability. While more complex designs, such as circular photonic crystal structures (e.g., photonic crystal disk lasers [39]), can achieve a better balance between sensitivity and *Q*-factor, our goal was to demonstrate that even with minimal structural optimization, the microdisk resonator could maintain a sufficient *Q*-factor for lasing while enhancing light–matter interaction. This trade-off prioritizes practical fabrication and scalability while still delivering measurable performance improvements.

**Figure 2:**
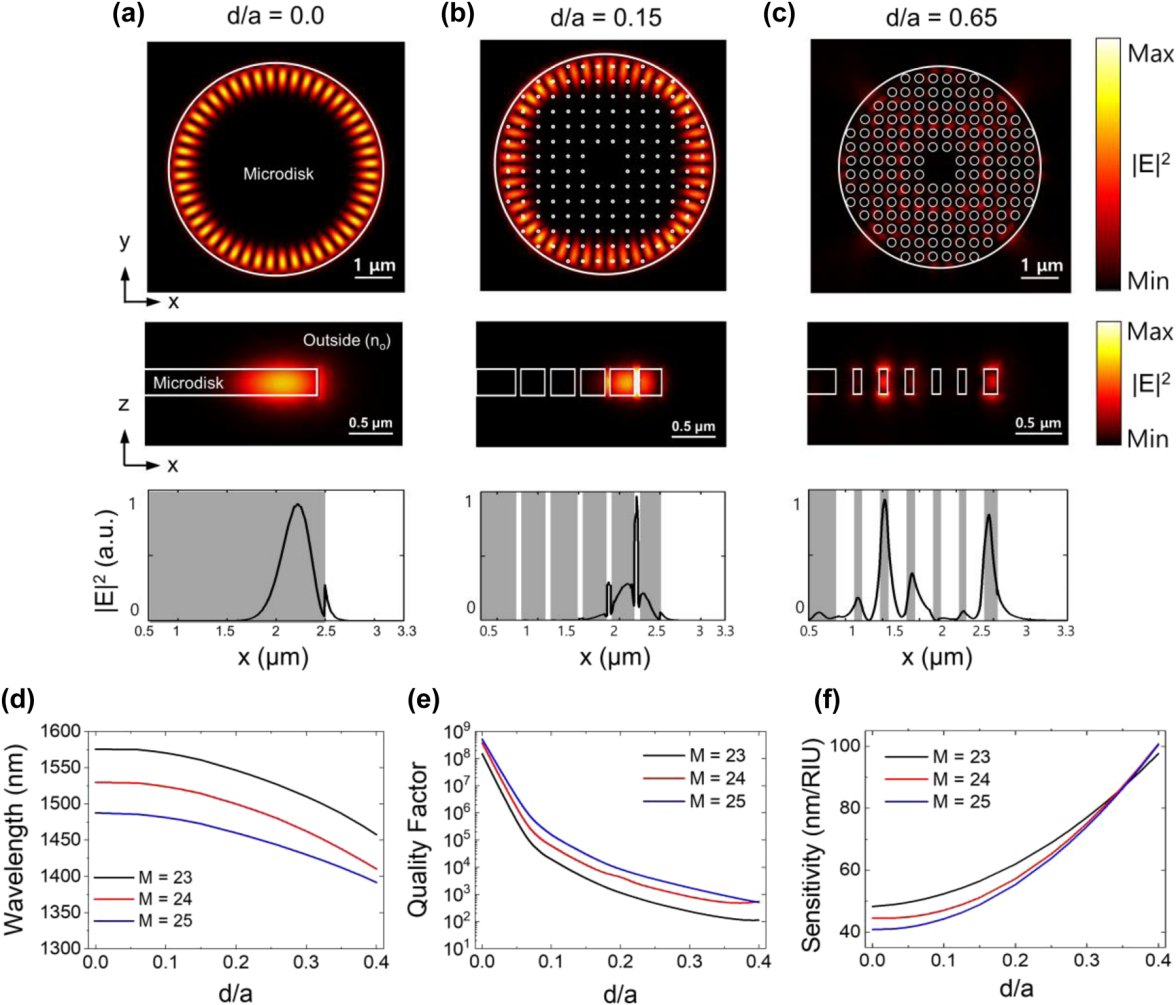
Simulated characteristic analysis of meta-hole patterned microdisk laser sensor. (a–c) |*E*|^2^ distributions of the meta-hole patterned microdisk laser cavities with *d*/*a* ratios of 0.0, 0.15, and 0.65 in the *xy*-plane, *xz*-plane, and along the *x*-axis from the center of the microdisk. The lattice constant *a* is fixed at 340 nm, and the WGM is presented for mode number *M* = 24. (d–f) Calculation results for resonance wavelength (*λ*), quality factor (*Q*-factor), and sensitivity (Δ*λ*/Δ*n*_0_) as a function of *d*/*a* for WGM mode numbers *M* = 23, 24, and 25. For *d*/*a* values exceeding 0.4 (*d* > 136 nm), no significant WGM mode is observed in all three modes.

Building on this design concept, finite element method (FEM) simulations were conducted to quantitatively analyze the characteristics of the microdisk resonant modes and their sensitivity as the size of the meta-holes was varied (see Section 5). Figure 2a–c shows the field distribution of the resonant mode (transverse electric (TE) mode) near *λ* = 1,500 nm for different meta-hole size ratios (*d*/*a* = 0.0, 0.15, 0.65) when the surrounding medium is methanol at room temperature (*n*_0_ = 1.3314). The figures illustrate the electric field intensity (|*E*|^2^) distributions across both the *xy* and *xz* cross sections, as well as along the *x*-axis from the center of the disk. Figure 2a illustrates the resonant mode of a microdisk without meta-holes, where the mode number (*M*) is 24, resonating at a wavelength of 1,529.9 nm. In this configuration, the quality factor (*Q*-factor) is calculated to be 3.8 × 10^8^. As illustrated, the majority of the energy of this original resonant mode is confined within the microdisk, with only 12 % of the total energy interacting with the external medium (see Supplementary Information). This energy proportion arises from the evanescent field extending into the external medium, a characteristic influenced by the thin microdisk thickness and the WGM mode properties, as observed in the field distribution along the *xz*-cross section and the electric field diagram along the *x*-axis. Figure 2b displays the |*E*|^2^ distribution for a microdisk with very small meta-holes (*d* = 51 nm, *d*/*a* = 0.15). Despite the introduction of small metal-holes, the WGM retains its overall shape, while the field becomes more concentrated in the meta-hole regions, as observed in the field distribution along the *xz*-cross section and the electric field diagram along the *x*-axis. Through this interaction, the WGM mode engages with the meta-hole regions, enhancing the overlap with the external medium and increasing the mode’s sensitivity to external refractive index changes. These subwavelength meta-holes reduce the effective refractive index of the resonator through interactions with the external medium, thereby enhancing sensitivity to external analytes without significantly disrupting the original resonant mode (see Supplementary Information). In this case, the resonant wavelength blue-shifts to 1,514.5 nm, and the *Q*-factor drops to 1.2 × 10^4^. The decrease in *Q*-factor is due to weaker WGM confinement, resulting from the reduced effective refractive index, and additional losses caused by Rayleigh scattering from the meta-holes [37]. While the *Q*-factor affects the lasing threshold power, once the pump power exceeds this threshold, the linewidth is primarily determined by stimulated emission, making the *Q*-factor less crucial for emission or sensing linewidth [40]. Thus, the primary concern is achieving resonance conditions with a sufficient *Q*-factor to allow lasing. When the hole size approaches the wavelength of light inside the laser material (*λ*/*n* ∼ 440 nm), the holes no longer behave as meta-holes and begin to strongly disrupt the resonant mode. Figure 2c shows the resonance mode distribution near a wavelength of 1,500 nm when the hole diameter is increased to 220 nm (*d*/*a* = 0.65). In this case, the WGM is no longer sustained, and only a weakly confined, atypical mode is observed. This structure loses its function as a WGM sensor, and the *Q*-factor drops to the tens, far below the threshold required for lasing.

Figure 2d–f presents the comprehensive results for the variations in resonant wavelength (*λ*), *Q*-factor, and sensitivity (Δ*λ*/Δ*n*_0_) for resonant modes 23, 24, and 25, respectively, as the meta-hole size ratio (*d*/*a*) increases. Our simulations indicate that for *d*/*a* values exceeding 0.4 (*d* > 136 nm, approximately 0.3 *λ*/*n*), no significant WGM modes were observed, prompting us to focus our analysis on *d*/*a* values below 0.4. As the meta-hole size increases across all three modes, both the resonant wavelength and *Q*-factor decrease, while sensitivity to refractive index changes improves. For resonant modes 23, 24, and 25, the resonant wavelengths decrease from 1,575.6 nm, 1,529.9 nm, and 1,487.7 nm to 1,457.6 nm, 1,410.3 nm, and 1,391.6 nm, respectively, as *d*/*a* increases from 0 to 0.4. At the same time, the *Q*-factor drops sharply from 1.5 × 10^8^, 3.8 × 10^8^, and 5.2 × 10^8^ to 1.1 × 10^2^, 5.5 × 10^2^, and 5.0 × 10^2^. Notably, mode 24 maintains a *Q*-factor above 10^3^ up to a *d*/*a* ratio of 0.3 and closely aligns with the 1,500 nm wavelength – the gain region of the chosen laser medium – making it the most promising candidate for laser operation. In contrast to the decreasing *Q*-factor, sensitivity increases from 48.2 nm/RIU, 44.5 nm/RIU, and 40.8 nm/RIU for modes 23, 24, and 25, respectively, to 97.7 nm/RIU, 100.8 nm/RIU, and 100.5 nm/RIU, representing 2.02-fold, 2.26-fold, and 2.46-fold improvements, respectively. This substantial enhancement in sensitivity is attributed to the increasing meta-hole size. Mode 24, with a central wavelength near 1,500 nm, stands out as the most promising for laser operation, striking an optimal balance between maintaining a high *Q*-factor and achieving the highest sensitivity among the modes. Interestingly, the sensitivity of mode 24 increases as *a* decreases (see Supplementary Information); however, in this study, *a* was fixed at 340 nm to ensure the feasibility of fabricating the small meta-holes. At *d*/*a* = 0.4, mode 24 demonstrates that 25 % of its resonant mode energy interacts with the external medium, further underscoring its superior performance as a laser sensor.

## 3 Experiment

**Figure 3:**
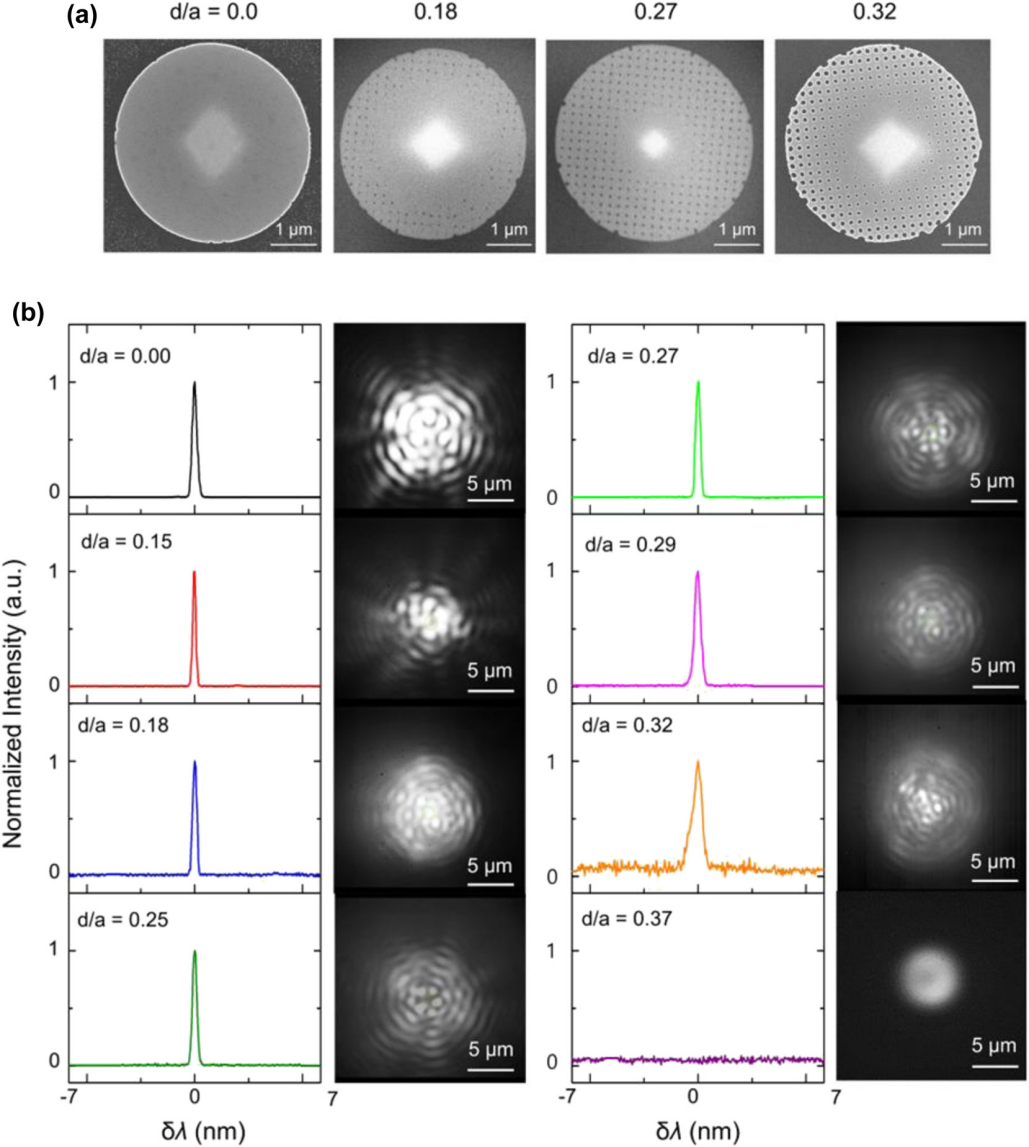
Experimental characterization of meta-hole patterned microdisk laser. (a) Scanning electron microscope (SEM) images of fabricated microdisk lasers with meta-hole patterns for *d*/*a* ratios of 0, 0.18, 0.27, and 0.32, with *d*/*a* values estimated from the SEM images. (b) Emission spectra of the meta-hole patterned microdisk lasers with *d*/*a* ratios of 0, 0.15, 0.18, 0.25, 0.27, 0.29, 0.32, and 0.37, with peak wavelengths centered for comparison, accompanied by IR CCD images of the laser emissions. The pump beam diameter was 7 μm, and the average pump power was maintained at 400 μW.

Based on the simulation results, we fabricated microdisk lasers with patterned meta-holes using electron beam lithography and chemically assisted ion beam etching (CAIBE) techniques, as shown in Figure 3a (see Section 5 and Supplementary Information). To systematically assess the sensor’s performance with varying meta-hole sizes, we incrementally increased the *d*/*a* ratio from 0 to 0.4. The meta-holes were uniformly spaced at 340 nm, although some variation in hole size was observed due to the proximity effect of electron beam lithography. We calculated the *d*/*a* ratio from scanning electron microscope (SEM) images of the fabricated structures (see Supplementary Information). Additionally, no meta-holes were introduced near the center of the microdisk to leave a supporting pedestal during the wet-etching process.

The performance of the microdisk lasers with varying meta-hole sizes was evaluated by placing them in a liquid injection chamber (see Section 5 and Supplementary Information). Methanol (99.5 % purity), with a refractive index of 1.3314, was injected, and laser performance was measured using a photoluminescence (PL) system with the following pump beam settings: wavelength = 976 nm, average power = 400 μW, pulse frequency = 1 MHz, pulse width = 50 ns, and beam diameter ∼7 μm (see Section 5 and Supplementary Information). Figure 3b displays the emission spectra of the meta-hole patterned microdisk lasers, with the peak wavelengths centered for comparison, alongside IR CCD images of the laser emission. As illustrated, all samples with *d*/*a* ≤ 0.32 demonstrated successful laser operation. Notably, all laser emissions occurred at a single wavelength across a wide spectral range of 1,400–1,700 nm (see Supplementary Information). The mode number of the laser emission was estimated to range from *M* = 22 to *M* = 24, depending on the overlap between the resonant wavelength and the gain region, which was influenced by the *d*/*a* ratio. The axial symmetry of the emission images confirmed that the WGM symmetry was preserved, and interference fringes in IR CCD images further verified the coherence of the laser beam. Variations in emission images with different *d*/*a* ratios were likely due to scattering effects induced by the meta-holes.

We also observed a sharp increase in the laser threshold power as the *d*/*a* ratio exceeded approximately 0.20 (see Supplementary Information), which can be attributed to a substantial reduction in the *Q*-factor. Theoretically, the *Q*-factor decreases continuously as *d*/*a* increases, as discussed in Figure 2e; however, in the fabricated samples, the disk roughness and irregularity of the meta-holes lowered the *Q*-factor even in structures without meta-holes, making the *Q*-factor reduction less pronounced at smaller *d*/*a* values. Beyond *d*/*a* > 0.20, however, the further reduction in the *Q*-factor appears to result in an increase in the threshold power. This increase in threshold power caused broadening of the laser linewidth at fixed pump power (see Supplementary Information). For *d*/*a* ratios of 0.29 and below, the linewidth (full width at half maximum) remained under 0.5 nm but increased to 0.65 nm at *d*/*a* = 0.32. At *d*/*a* = 0.37, no laser peak was observed, and the emission image resembled spontaneous emission, indicating the cessation of laser operation. This was due to the *Q*-factor being reduced to a level insufficient to meet the lasing conditions.

**Figure 4:**
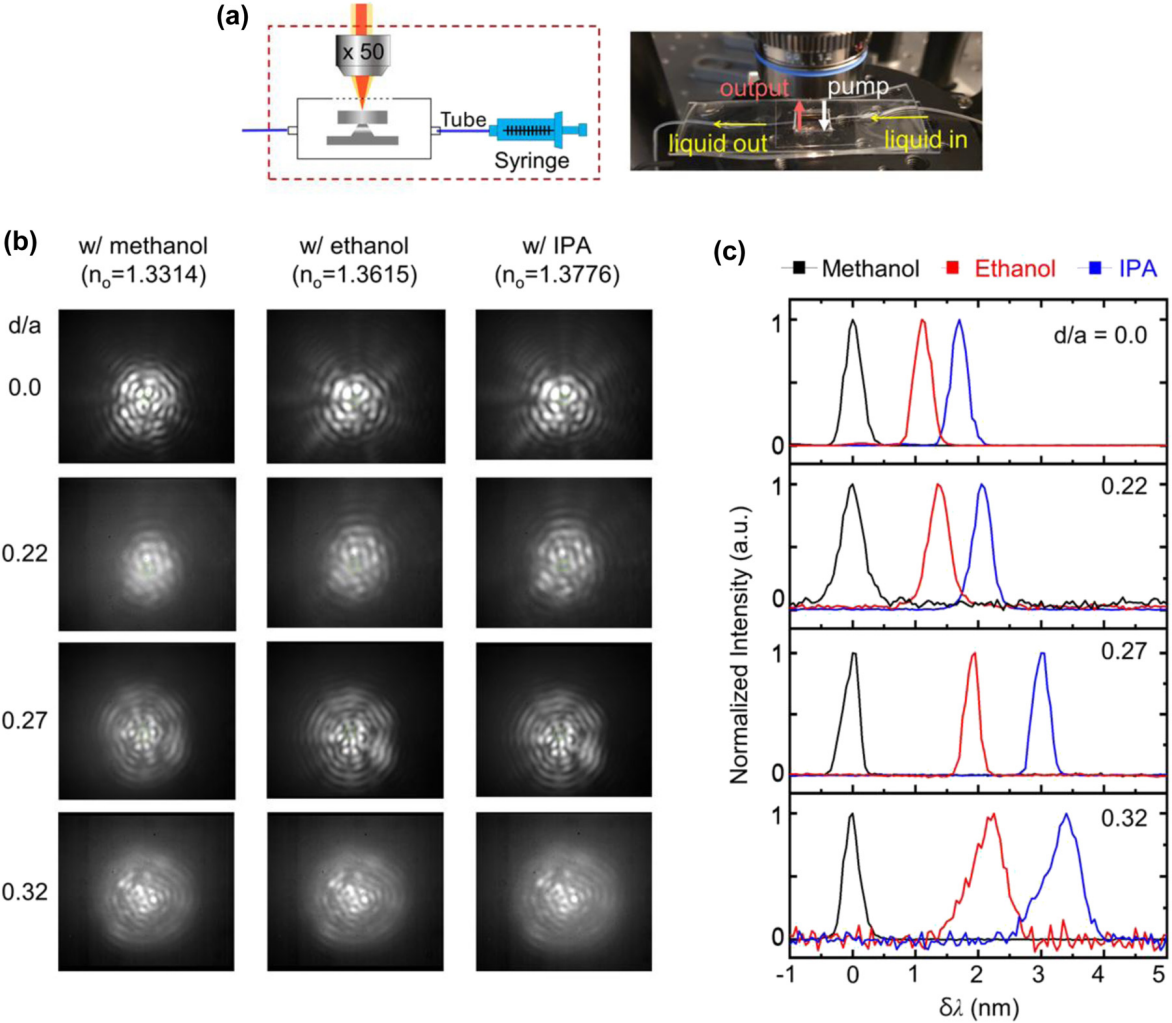
Refractive index sensing performance of meta-hole patterned microdisk laser sensor with various analytes. (a) Chamber setup for assessing the sensitivity of the fabricated microdisk laser sensor to different injected liquids: methanol (99.5 %, *n*_0_ = 1.3314), ethanol (94.5 %, *n*_0_ = 1.3615), and isopropyl alcohol (IPA) (99.5 %, *n*_0_ = 1.3776), sequentially introduced through a silicone tube. (b) Emission images of microdisk lasers with *d*/*a* ratios of 0.0, 0.22, 0.27, and 0.32 as the surrounding medium is sequentially changed to methanol, ethanol, and IPA. (c) Spectral response of microdisk sensors with various *d*/*a* ratios as the surrounding liquid changes.

To evaluate the sensitivity of the fabricated microdisk laser sensor to different injected liquids, we used a chamber setup as shown in Figure 4a. Methanol (99.5 %), ethanol (94.5 %), and isopropyl alcohol (IPA) (99.5 %) were introduced sequentially through a silicone tube at a flow rate of 0.1 mL/min. Once the spectrum and emission images stabilized, the flow was halted for measurements (see Section 5). The refractive indices of each liquid, measured precisely beforehand for comparison, were 1.3314 for methanol, 1.3615 for ethanol, and 1.3776 for IPA.

The first objective was to examine whether the emission profile of the microdisk laser, particularly the WGM mode, was maintained across different liquids for microdisks with varying meta-hole sizes. Figure 4b presents the emission images for microdisk lasers with *d*/*a* ratios of 0.0, 0.22, 0.27, and 0.32, as the surrounding medium was sequentially changed to methanol, ethanol, and IPA. All images displayed a WGM-like emission profile with strong interference fringes, indicating stable laser operation. Furthermore, for a given *d*/*a* ratio, the emission profile remained consistent across different liquids. To quantify this consistency, we calculated the structural similarity index (SSIM) for the emission images across different liquids, finding that all configurations maintained an SSIM above 0.75, indicating a high degree of image similarity, regardless of liquid type (see Supplementary Information).

Figure 4c shows the spectral response of microdisk sensors with various *d*/*a* ratios as the surrounding liquid changed. For this measurement, the pump average power was fixed at 400 μW. The data indicate that as the meta-hole size increases (i.e., as *d*/*a* rises), the resonance wavelength shift due to the subtle refractive index differences of the liquids becomes more pronounced. For a microdisk without meta-holes, the resonance wavelengths in ethanol and IPA environments shifted by 1.11 nm and 1.70 nm, respectively, relative to the resonance in methanol. In contrast, for the microdisk with the largest meta-hole (*d*/*a* = 0.32), these shifts were 2.20 nm and 3.44 nm, corresponding to sensitivity increases of 1.98-fold and 2.02-fold, respectively. The laser emission linewidth also exhibited notable trends: for *d*/*a* ratios up to 0.27, the laser linewidth (FWHM) remained below 0.45 nm, showing minimal variation across different liquids. However, for *d*/*a* = 0.32, the linewidth broadened to over 0.6 nm in ethanol and IPA environments. This broadening is attributed to the increase in laser threshold power associated with the higher refractive indices of the surrounding liquids.

**Figure 5:**
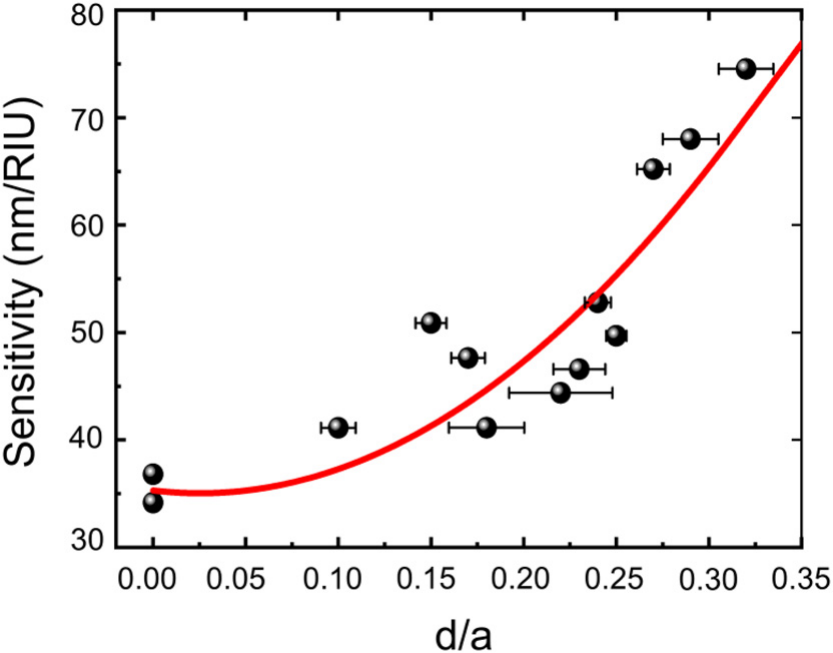
Sensitivity analysis of meta-hole patterned microdisk laser sensor. A comprehensive graph displays sensor sensitivity as a function of the *d*/*a* ratio, showing a nonlinear quadratic increase in sensitivity with increasing meta-hole size, closely aligning with theoretical predictions. The red curve represents a quadratic polynomial fit to the measured data points.

Figure 5 provides a comprehensive graph showing sensor sensitivity as a function of the *d*/*a* ratio, with a nonlinear quadratic increase in sensitivity as the meta-hole size increases, closely matching theoretical predictions (see Supplementary Information). The red curve represents a quadratic polynomial fit to the measured data points. As shown, when the *d*/*a* ratio exceeds 0.25, sensitivity rises sharply above 60 nm/RIU, reaching a peak of 74.5 nm/RIU at *d*/*a* = 0.32. This peak represents a 2.02-fold increase compared to the 36.8 nm/RIU sensitivity observed in the structure with *d*/*a* = 0. The variations in measured sensitivity among samples with the same *d*/*a* ratio are likely due to factors such as inconsistencies in meta-hole uniformity and edge irregularities of the microdisk caused by fabrication process errors.

## 4 Discussion & conclusion

We have introduced a novel approach to enhancing the sensitivity of microdisk laser sensors by incorporating periodic meta-hole patterning, successfully overcoming the limitations of conventional microdisk designs. The addition of meta-holes facilitated increased interaction with external analytes while preserving the WGM profile, resulting in significantly improved sensitivity. Through simulations and experimental validation, we achieved a maximum sensitivity of 100.8 nm/RIU in simulation – a 2.26-fold increase compared to nonpatterned microdisks – and an experimentally verified sensitivity of 74.5 nm/RIU at a *d*/*a* ratio of 0.32, reflecting a 2.02-fold improvement.

Despite these advancements, our approach encountered several challenges. Achieving uniform meta-hole fabrication remains difficult due to lithographic variability, impacting sensor performance and reproducibility. Additionally, while periodic patterning enhances sensitivity, it introduces a trade-off with the *Q*-factor, particularly as meta-hole sizes approach the effective wavelength scale. Managing this balance is crucial since a reduced *Q*-factor can elevate the lasing threshold and affect signal stability. To address these limitations further, exploring alternative designs or hybrid materials may enhance performance across a broader wavelength range or in diverse environments, thus increasing sensor versatility. Additionally, optimizing meta-hole geometry could preserve a high *Q*-factor even with larger holes, allowing for greater analyte interaction without compromising stability.

In conclusion, this study advances miniaturized, high-performance optical sensors, presenting a promising approach for applications in biological, environmental, and chemical sensing. With continued progress in both design and fabrication, meta-hole patterned microdisks hold significant potential for integration into next-generation photonic systems, paving the way for highly sensitive, robust sensors capable of meeting real-world demands.

## 5 Methods

### 5.1 Numerical simulations

We employed the finite element method (FEM) using COMSOL Multiphysics (version 5.4) to compute the electric field distribution, *Q*-factor, and resonance wavelength of the proposed meta-hole patterned microdisk laser cavity. The microdisk was designed with a 5 μm diameter and a 250 nm thickness, while the pedestal was modeled as a rectangular column with an 800 nm width and 1 μm height. The refractive indices were set to InGaAsP for the microdisk (*n* = 3.45), InP for the pedestal (*n* = 3.12), and varied between 1 and 1.5 for the surrounding environment to simulate different external conditions. A tetrahedral mesh was applied, with separate element sizes for the meta-hole microdisk and surrounding regions. The mesh element sizes ranged from 40 nm to 100 nm within the microdisk, capturing the meta-holes’ details, while the surrounding regions used mesh sizes between 80 nm and 450 nm.

### 5.2 Fabrication of meta-hole patterned microdisk laser

The meta-hole patterned microdisk was fabricated using an InGaAsP wafer. The InGaAsP wafer consisted of a top layer of 250 nm thick InGaAsP with a multiquantum well structure, a 400 nm thick sacrificial InP layer, and an InP substrate. A 300 nm thick layer of 950 PMMA C4 was coated onto the wafer, and patterning was performed using electron beam lithography. Afterward, the wafer was heated to 453 K, and etching was conducted for 9 s using a chemically assisted ion beam (CAIBE) composed of Cl_2_ gas and Ar gas. The etching process reached through both the InGaAsP layer and the sacrificial InP layer. The remaining resist was removed by exposing the sample to O_2_ plasma at 30 sccm and 150 W for 20 min. Finally, the lower InP layer, excluding the pedestal structure supporting the microdisk, was removed by immersing the wafer in a solution of 35 % HCl and deionized water (3:1 ratio) for 1 min at room temperature.

### 5.3 Fabrication of liquid flow system and liquid exchange process

To effectively change the external refractive index of the meta-holes, we fabricated a liquid flow system. First, a thin layer of UV adhesive (Norland Optical Adhesive NOA 81) was applied to the back of the wafer containing the meta-holes using a razor blade or cover glass, and it was attached to a glass slide. The adhesive was cured using a UV lamp for approximately 10 min. To create the PDMS (DOW CORNING, Silgard Silicone Elastomer kit) chamber, silicone molding agents (Agent: Base = 1:10) were mixed, the bubbles were removed, and a 0.5 mm diameter silicone tube was inserted before curing at room temperature for about 7 days. The cured PDMS chamber was cut to match the size of the glass slide, and a hole with a diameter of approximately 5 mm was made to accommodate the sample containing the meta-holes. The PDMS chamber was then bonded to the slide glass, which already had the sample attached, after surface treatment using O_2_ plasma. One end of the silicone tube was connected to a syringe pump (Newera NE300) to control the liquid flow, and methanol, ethanol, and IPA were injected through a 1 mL syringe. The flow rate was set to 0.1 mL/min. As the total volume of the liquid system, including the silicone tube, was 3 mL, when changing the liquid, the system was flushed with twice the volume of the desired new liquid to ensure complete exchange.

### 5.4 Photoluminescence analysis

For laser excitation of the meta-hole patterned microdisk laser, a 976 nm laser (Thorlab, BL976-SAG300) was connected to a function generator (Keysight 33600A) to generate pulse waves with a frequency of 1 MHz and a pulse width of 50 ns. The generated pulse was delivered to the microdisk laser via a 50× magnification, N.A 0.65 IR objective lens (Olympus, LCPLN). The signal emitted from the microdisk laser was directed to an IR CCD (Goodrich-SU320HX-1.7RT) and a monochromator (Spectral Products, DK480) with a 1,200 g/mm grating through a 5:5 beam splitter, enabling the measurement of PL images and spectra.

## Supplementary Material

Supplementary Material Details
