# Supplementary material for: Highly sensitive microdisk laser sensor for refractive index sensing via periodic meta-hole patterning
Source: Nanophotonics. 2025 Jan 30;14(8):1193–202. doi: 10.1515/nanoph-2024-0598 (PMC12019935; doi:10.1515/nanoph-2024-0598)
Supplement: Supplementary file 1 — Supplementary Material Details [file j_nanoph-2024-0598_suppl_001.pdf]

Supplementary Information for

Highly sensitive microdisk laser sensor for refractive index sensing via periodic meta-hole patterning

Haerin Jeong[1], Nu-Ri Park[1], Byoung Jun Park[1], Moohyuk Kim[1], Jin Tae Kim[2], and Myung-Ki Kim[1,3*]*

[1] KU-KIST Graduate School of Converging Science and Technology, Korea University, Seoul 02841, Republic of Korea

[2] Quantum Technology Research Department, Electronics and Telecommunications Research Institute (ETRI), Daejeon, 34129, Republic of Korea

[3] Center for Quantum Information, Korea Institute of Science and Technology (KIST), Seoul 02792, Republic of Korea

[*]Corresponding authors. Email: jintae@etri.re.kr (J. T. K.), rokmk@korea.ac.kr (M.-K. K.)

Contents

1. Effective Refractive Index of Microdisk Resonator with Meta-Holes
2. Resonant Condition and Sensitivity of Microdisk Resonator Sensor with Meta-Holes
3. Energy Proportion Outside the Meta-Holes Patterned Microdisk Resonator
4. Sensor Performance with Different Hole Periodicities
5. Fabrication of Meta-Hole Patterned Microdisk Laser
6. Estimation of d/a Ratios from SEM Image Analysis
7. Fabrication of Fluidic Chamber
8. Experimental Setup
9. Laser Spectra Across a Wide Spectral Range
10. Measured Laser Threshold Powers and Linewidths
11. Structural Similarity Index (SSIM) Calculation

Supplementary Note 1: Effective Refractive Index of Microdisk Resonator with Meta-Holes

The effective refractive index (n_{eff}) of a microdisk resonator incorporating meta-holes (refractive index = n_{in}) is primarily determined by two factors. The first factor, *γ*, arises from the overlap between the evanescent decay field, influenced by the finite thickness and the whispering gallery mode (WGM) characteristics, and the external material (refractive index = n_{out}). The second factor, *f*, arises from the influence of the meta-holes, which replace the original resonator material (n_{in}) with the external material (n_{out}) within the meta-holes, which are much smaller than the wavelength. The effective refractive index of the resonant mode, incorporating these effects, can be expressed as:

$$n_{eff} = n_{in} + \gamma \cdot (n_{out} - n_{in}) + f \cdot \left(n_{out} - n_{in}\right) \tag{S1}$$

Here, *γ* is determined by the decay depth of the evanescent field and typically increases as the thickness decreases or as the disk radius R reduces. *f* is defined by the ratio of the volume occupied by the meta-holes to the total volume of the microdisk.

Under resonant conditions, *γ* remains constant, while *f* depends on the size and numbers of the meta-holes. For a disk with radius R and thickness *t*, where meta-holes with diameter *d* are periodically arranged with spacing *a*, the approximate number of meta-holes, N, can be expressed as $N = (2R/a)^2$. Consequently, *f* is calculated as:

$$f = \frac{V_{holes}}{V_{disk}} = \frac{N\pi(d/2)^2 t}{\pi R^2 t} = \left(\frac{2R}{a}\right)^2 \frac{\left(d/2\right)^2}{R^2} = \left(\frac{d}{a}\right)^2 \tag{S2}$$

Substituting this into Eq. (S1), the effective refractive index is expressed as:

$$n_{eff} = n_{in} + \left(n_{out} - n_{in}\right) \cdot \gamma + \left(n_{out} - n_{in}\right) \cdot \left(\frac{d}{a}\right)^2 \tag{S3}$$

Supplementary Note 2: Resonant Condition and Sensitivity of Microdisk Resonator Sensor with Meta-Holes

The condition for whispering gallery modes (WGMs) in a microdisk resonator is given by:

$$M \cdot \lambda_M = 2\pi R \cdot n_{eff} \tag{S4}$$

where M is the mode number, λ_M is the resonant wavelength, R is the disk radius, and n_{eff} is the effective refractive index. Substituting Eq. (S3) into this condition gives:

$$\lambda_M = \frac{2\pi R}{M} \cdot \left[n_{in} + \left(n_{out} - n_{in}\right) \cdot \gamma + \left(n_{out} - n_{in}\right) \cdot \left(\frac{d}{a}\right)^2 \right] \tag{S5}$$

The sensitivity, defined as $\Delta\lambda_M/\Delta n_{out}$, is then given by:

$$sensitivity = \frac{\Delta\lambda_M}{\Delta n_{out}} = \frac{2\pi R}{M} \cdot \left[\gamma + \left(\frac{d}{a}\right)^2 \right] \tag{S6}$$

This shows a quadratic dependence of sensitivity on *d*/*a*, highlighting the significant role of the periodic meta-hole structure in enhancing the resonator's sensitivity.

Supplementary Note 3: Energy Proportion Outside the Meta-Hole Patterned Microdisk Resonator

The proportion of energy interacting with the external medium, $\eta_{outside}$, relative to the total energy of the resonant mode, was calculated using Eq. (S7), where ε represents the permittivity at each point in the system, and $\varepsilon_{outside}$ denotes the permittivity of the material outside the resonator. Figure S1 shows the calculated $\eta_{outside}$ as a function of d/a for the M = 24 mode of the microdisk resonator with a diameter of 5 μm at different thicknesses (t). The results indicate that $\eta_{outside}$ increases at a similar rate as d/a increases across all thicknesses. Additionally, as the thickness increases, the energy of the resonant mode becomes more confined within the resonator material, resulting in a decrease in $\eta_{outside}$. Conversely, as t decreases, the quality factor (Q-factor) degradation caused by the meta-holes becomes more pronounced. To balance these effects, the thickness was fixed at t = 250 nm in this study. Under this condition, the energy proportion $\eta_{outside}$ was calculated to be 12% at $d/a = 0$ and 25% at $d/a = 0.4$, respectively.

$$\eta_{outside} = \frac{\int_{outside} \varepsilon_{outside} |E|^2 dV}{\int_{total} \varepsilon |E|^2 dV} \tag{S7}$$

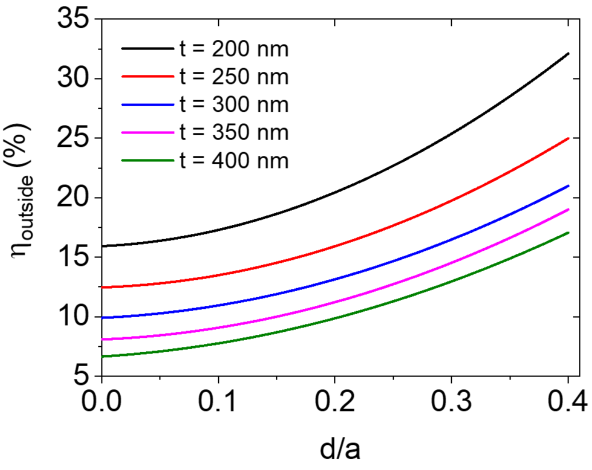

Fig. S1. Energy proportion outside the resonator. Calculated energy proportion interacting with the external material $\eta_{outside}$ as a function of d/a for the M = 24 mode of a microdisk resonator with a diameter of 5 μm at different thicknesses (t). The red line indicates the fixed thickness t = 250 nm used in this study, where $\eta_{outside}$ reaches 12% at d/a = 0 and 25% at d/a = 0.4.

Supplementary Note 4: Sensor Performance with Different Hole Periodicities

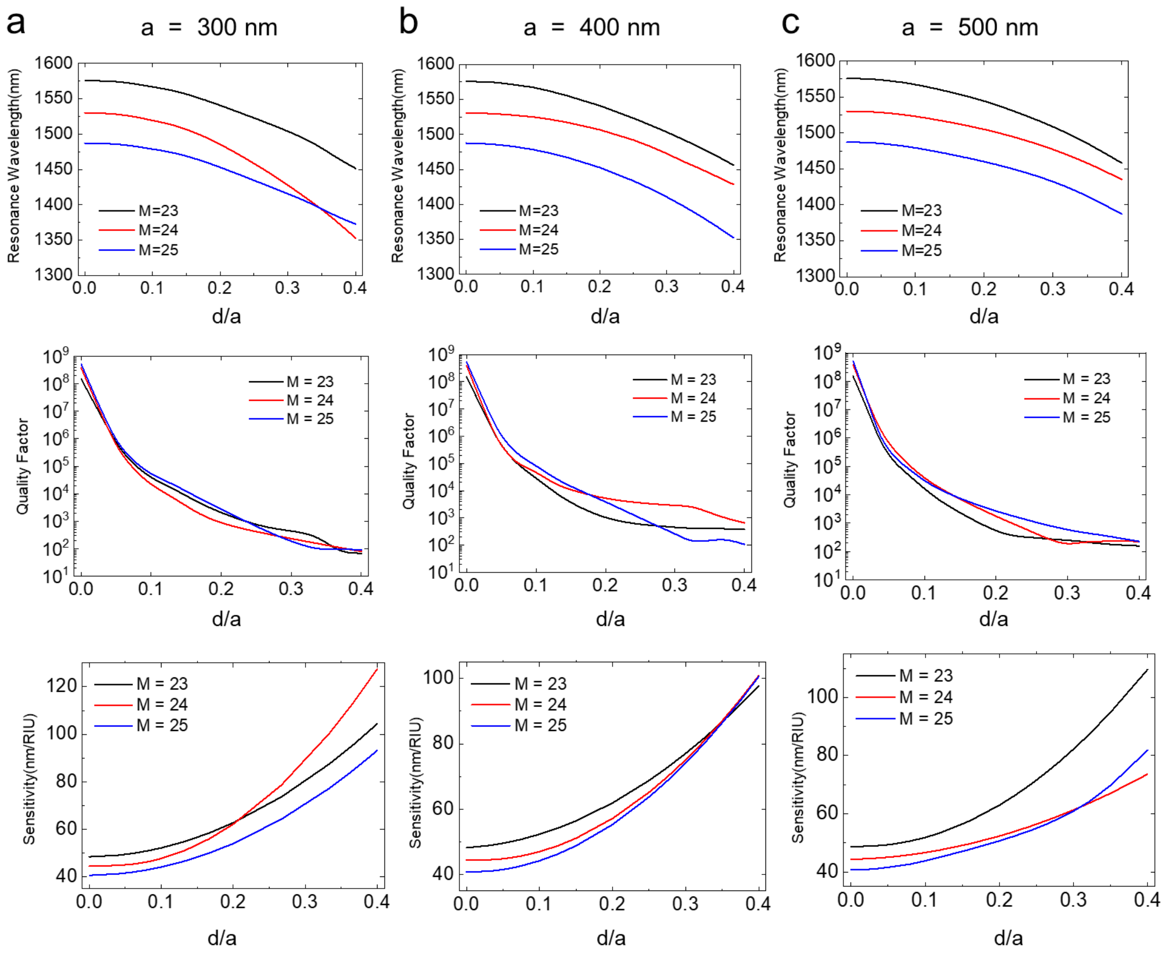

Fig. S2. Sensor performance with different meta-hole periodicities. (a-c) Variation in resonance wavelength, quality factor, and sensitivity of the meta-hole patterned microdisk laser cavity as a function of the *d*/*a* ratio for resonance modes 23, 24, and 25, with periodicities *a* of 300 nm, 400 nm, and 500 nm, respectively. In all three periodicity structures, increasing the *d*/*a* ratio leads to a decrease in both resonance wavelength and quality factor, accompanied by an increase in sensitivity. The trends are consistent across the three cases, with similar numerical values observed. For the target mode 24, sensitivity consistently improves as the periodicity decreases. Considering the feasible dimensions for fabricating the meta-holes, a periodicity of 340 nm was selected for this study.

Supplementary Note 5: Fabrication of Meta-Hole Patterned Microdisk Laser

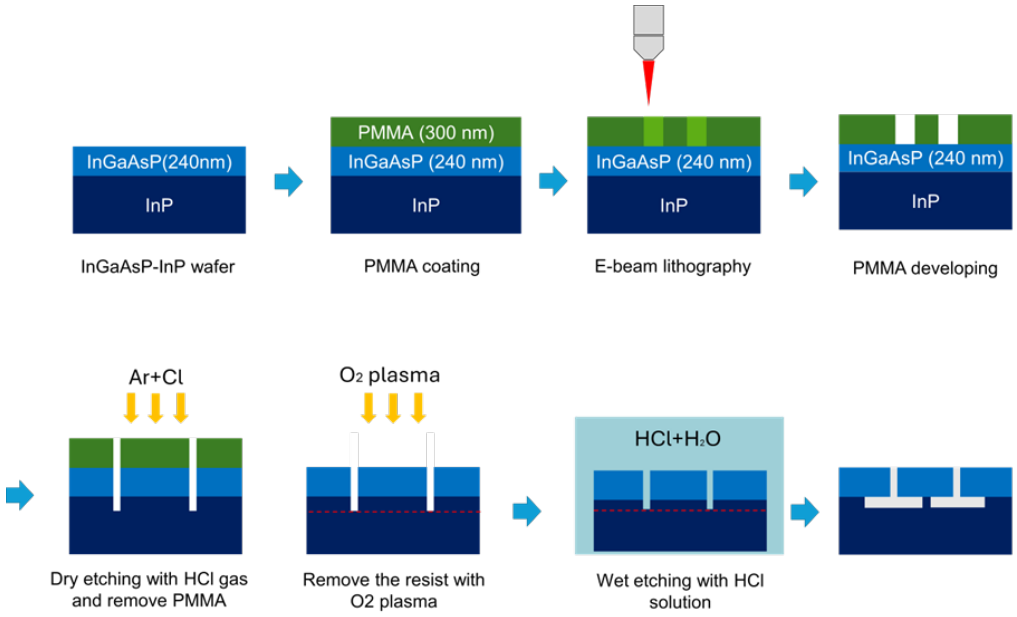

Fig. S3. Fabrication process of meta-hole patterned microdisk laser. The meta-hole patterned microdisk was fabricated using an InGaAsP wafer, consisting of a 250 nm-thick InGaAsP top layer with a multi-quantum well structure, a 400 nm-thick sacrificial InP layer, and an InP substrate. A 300 nm-thick layer of 950 PMMA C4 was spin-coated onto the wafer, and electron beam lithography was employed to define the meta-hole patterns. The wafer was then heated to 453 K, followed by a 9-second chemically-assisted ion beam etching (CAIBE) process using Cl_2 and Ar gases, which etched through both the InGaAsP and sacrificial InP layers. The residual resist was removed by O_2 plasma treatment at 30 sccm and 150 W for 20 minutes. To form a stable pedestal structure supporting the microdisk, a central region measuring approximately 800 nm x 800 nm was left unpatterned during lithography. In the final wet etching step, the lower InP layer, excluding the pedestal, was selectively removed by immersing the wafer in a solution of 35% HCl and deionized water (3:1) for around 45 seconds at room temperature. The immersion time was carefully controlled to accurately define the pedestal height and prevent over-etching, with the optimal etching duration established through preliminary experiments to ensure consistent pedestal formation across samples.

Supplementary Note 6: Estimation of *d*/*a* Ratios from SEM Image Analysis

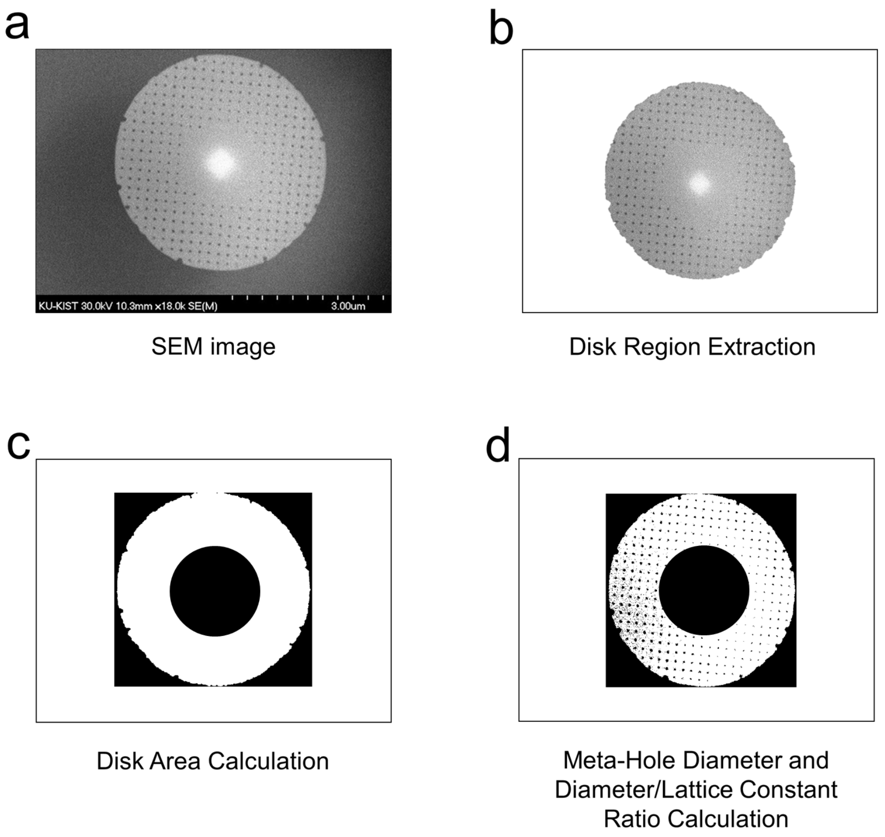

Fig. S4. Estimation of *d*/*a* Ratios from SEM Image Analysis. (a) SEM Image: SEM image of a microdisk laser with a uniform array of meta-holes. (b) Disk Region Extraction: The region of interest, the microdisk, is isolated from the SEM image, removing the background to focus exclusively on the meta-hole pattern. (c) Disk Area Calculation: The total disk area within a 1.5 μm inner region from the microdisk edge is calculated by converting the disk region to white, assigning a value of 1 to each white pixel and 0 to each black pixel, and summing these pixel values. (d) Meta-Hole Diameter and *d*/*a* Ratio Calculation: By applying an appropriate threshold, the meta-hole regions are converted to black while the remaining disk regions stay white. The diameter of each meta-hole is estimated based on pixel counts, and the lattice constant (spacing between holes) is measured in a similar manner. The *d*/*a* ratio, representing the diameter-to-lattice constant ratio, is then calculated for each hole, with an average *d*/*a* ratio determined for the entire pattern within the 1.5 μm inner region from the microdisk edge.

Supplementary Note 7: Fabrication of Fluidic Chamber

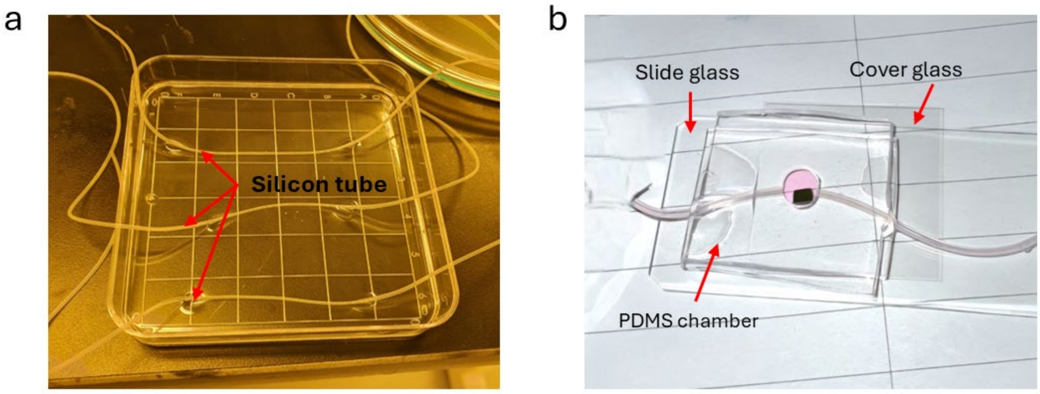

Fig. S5. Fabrication and assembly of the fluidic chamber. (a) Silicone tubes are embedded within uncured PDMS to create fluid channels. (b) The fluidic chamber comprises a slide glass base, PDMS walls, and an O_2 plasma-treated cover glass to ensure a sealed system. Fluids flow through the silicone tubes, while a central hole provides access to the sample.

A specialized fluidic chamber was constructed to enable experiments in various liquid environments. First, the wafer containing the meta-hole patterned microdisk lasers was adhered to a glass slide using a thin layer of UV adhesive (Norland Optical Adhesive NOA 81), which was applied to the back of the wafer and cured under a UV lamp for 10 minutes to ensure a secure bond. To create the PDMS chamber, silicone agents were mixed in a 1:10 ratio (curing agent to base), then degassed in a vacuum desiccator to remove air bubbles, and a 0.5 mm diameter silicone tube was embedded. The PDMS was allowed to cure at room temperature over 7 days. After curing, the PDMS was cut to match the glass slide and a 5 mm hole was created to align with the sample area. The PDMS chamber was then bonded to the glass slide with the sample using O_2 plasma treatment to form a leak-proof seal.

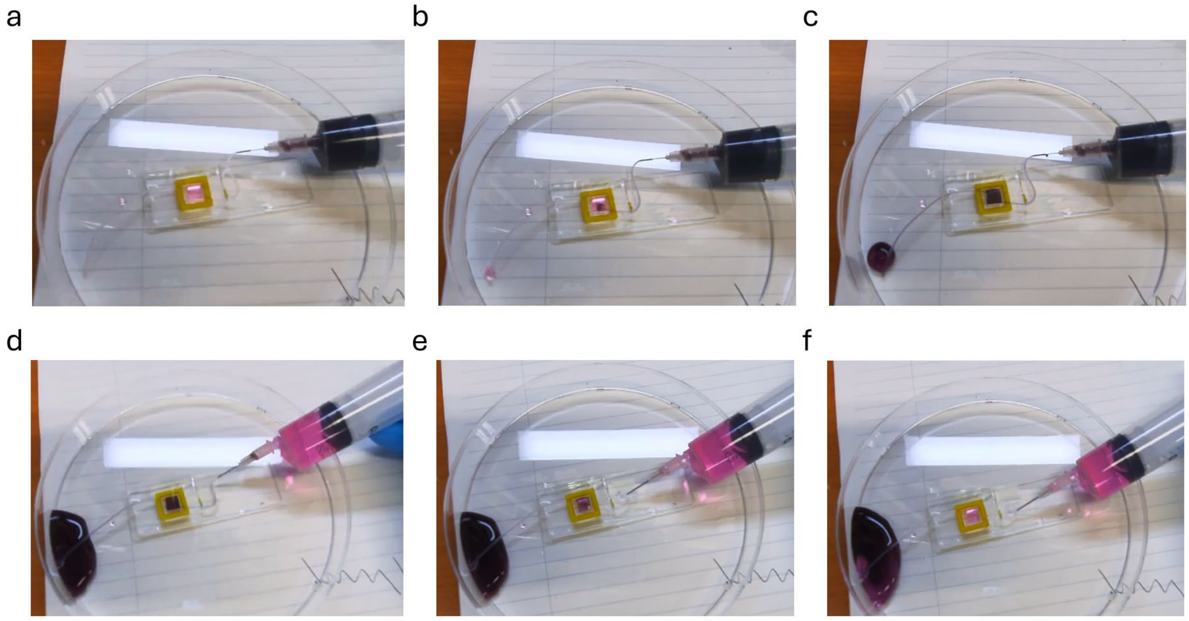

Fig. S6. Leakage testing and liquid replacement verification. (a) A pink liquid was initially injected to verify the chamber's seal integrity. (b) A black liquid was then introduced, visually illustrating the mixing process as the black liquid began displacing the pink. (c) Additional black liquid was injected to completely replace the pink, demonstrating a successful and thorough exchange without leakage. (d) In the reverse process, the chamber was initially filled with black liquid, and a bright pink liquid was introduced to initiate the exchange. (e) At the midpoint of the exchange, the pink liquid progressively replaced the black. (f) In the final stage, the pink liquid fully replaced the black, confirming a complete and successful exchange.

To assess the fluidic chamber's stability during extended use, leakage tests were conducted. First, a pink liquid was injected to check for leaks, and the liquid remained stable, confirming a secure seal. When black liquid was introduced into the chamber filled with pink liquid, the smooth mixing process demonstrated effective displacement of the original liquid. Finally, an excess of black liquid was injected to fully replace the pink liquid without any leakage. In the reverse scenario, complete replacement of black ink with pink ink was also confirmed, verifying the setup's capability for reliable and thorough liquid exchanges.

Supplementary Note 8: Experimental Setup

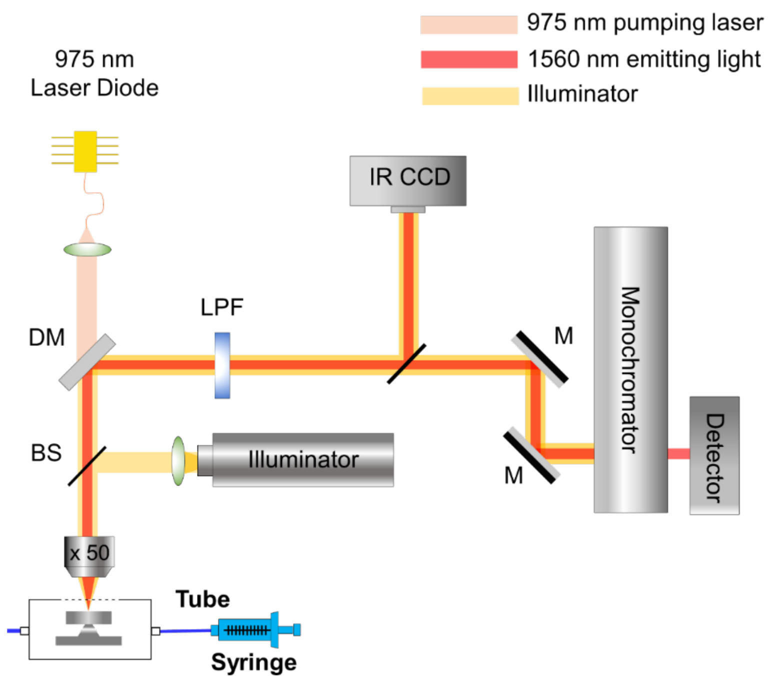

Fig. S7. Photoluminescence setup for laser characterization and liquid sensing. The meta-hole patterned microdisk laser was excited using a 976 nm laser (Thorlab, BL976-SAG300), controlled by a function generator (Keysight 33600A) to produce pulse waves at 1 MHz with a 50 ns pulse width. The laser beam was focused through a 50x magnification IR objective lens (Olympus, LCPLN, N.A. 0.65), creating a spot size of approximately 7 µm, and the laser power was maintained at 400 µW. The sample containing the microdisk laser was mounted on a motorized xyz stage with 30 nm resolution. Emission from the microdisk laser was collected through a 5:5 beam splitter and directed simultaneously to an IR CCD (Goodrich-SU320HX-1.7RT) and a monochromator (Spectral Products, DK480) with a 1200 g/mm grating, enabling simultaneous photoluminescence (PL) imaging and spectral analysis. To accurately position the microdisk laser, a tungsten halogen lamp was used for illumination. Data acquisition was performed at 128 Hz, and all experiments were conducted at room temperature.

Supplementary Note 9: Laser Spectra Across a Wide Spectral Range

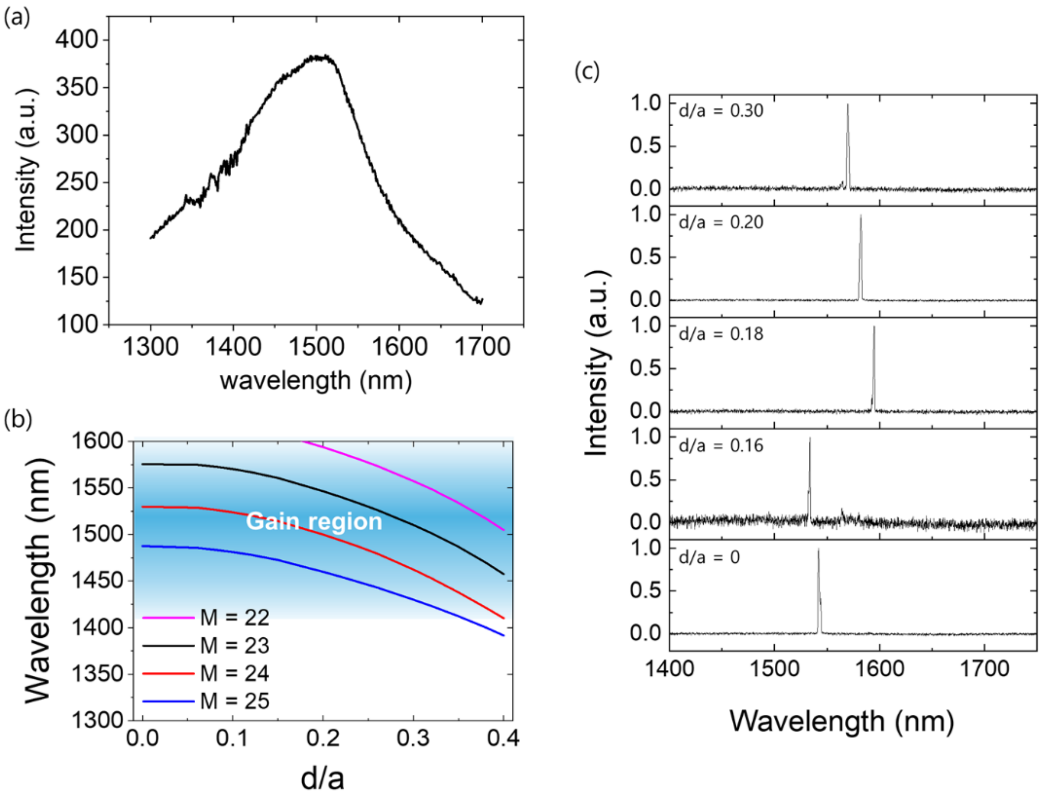

Fig. S8. Laser spectra across a wide spectral range. (a) Emission spectrum (gain spectrum) of the InGaAsP quantum well wafer used in this study, showing the spectral region where optical gain is achieved. (b) Overlap between the gain region and the wavelengths of the microdisk whispering gallery modes (WGMs) for different mode numbers (M = 22, 23, 24, 25) as a function of the normalized hole size (d/a). The shaded region indicates the spectral range of the gain region. (c) Laser spectra for various d/a values. Based on the results in (b), it is estimated that the mode at M = 23 is excited for the structures with $d/a = 0$ and $d/a = 0.16$, while the mode at M = 22 is excited for the structures with $d/a \geq 0.18$.

Supplementary Note 10: Measured Laser Threshold Powers and Linewidths

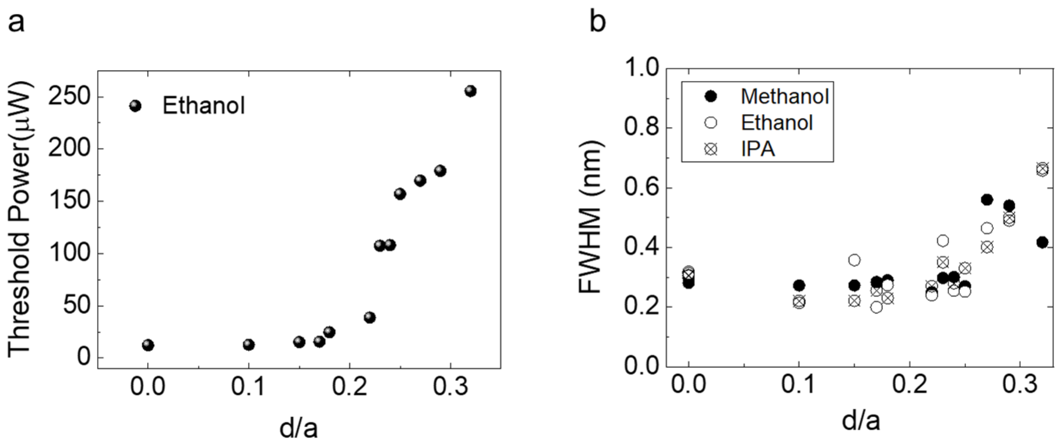

Fig. S9. Measured laser threshold powers and linewidths. (a) Threshold power as a function of the *d*/*a* ratio for the meta-hole patterned microdisk laser, measured in an ethanol environment. (b) Full width at half maximum (FWHM) of the laser spectral linewidth as a function of the meta-hole volume ratio, measured in methanol, ethanol, and IPA environments at a fixed pump average power of 400 μW.

Figure S9 illustrates the increase in threshold power and FWHM broadening with increasing *d*/*a* ratio for the meta-hole patterned microdisk laser. A marked rise in laser threshold power is observed as the *d*/*a* ratio exceeds approximately 0.20, attributed to a substantial reduction in the Q-factor. Theoretically, the Q-factor continuously decreases with increasing *d*/*a*, as shown in Fig. 2e. However, in fabricated samples, inherent disk roughness and meta-hole irregularities lowered the Q-factor even in structures without meta-holes, making the Q-factor reduction less pronounced at smaller d/a values. Beyond *d*/*a* > 0.20, further Q-factor reduction results in a noticeable increase in threshold power, causing additional broadening of the laser linewidth at fixed pump power. For *d*/*a* ratios of 0.29 and below, the linewidth remained under 0.5 nm but increased to 0.65 nm at *d*/*a* = 0.32.

Supplementary Note 11: Structural Similarity Index (SSIM) Calculation

The Structural Similarity Index (SSIM) is a metric used to assess the similarity between two images [S1, S2]. SSIM values range from -1 to 1, with 1 indicating that the two images are identical, values of 0.85 and above suggesting that the images are very similar, values between 0.7 and 0.85 indicating moderate similarity, values between 0.5 and 0.7 suggesting that the images are somewhat different, and values below 0.5 indicating that the images are significantly different

The SSIM calculation is based on three components: luminance, contrast, and structure. For two images, x and y, the SSIM index is calculated using the formula:

$$SSIM(x,y) = \frac{(2\mu_x\mu_y + C_1)(2\sigma_{xy} + C_2)}{(\mu_x^2 + \mu_y^2 + C_1)(\sigma_x^2 + \sigma_y^2 + C_2)}$$

In this formula, μ_x and μ_y represent the mean intensities of images x and y, while σ_x^2 and σ_y^2 represent the variances of x and y. The term σ_{xy} denotes the covariance of x and y. The constants C_1 and C_2 are small values added to stabilize the calculation, preventing instability when the denominator approaches zero. This approach enables SSIM to quantify the perceived quality of image similarity effectively.

To assess the consistency of the emission profiles in the microdisk laser across different refractive index environments (methanol, ethanol, and IPA), SSIM calculations were applied to PL images. The SSIM values provide insights into how the emission change as the microdisk is immersed in different liquids, helping to confirm if the same resonance mode is being maintained.

Table S1. SSIM values for emission images of samples with varying *d*/*a* ratios.

***d*/*a* ratio**	**Methanol vs. Ethanol**	**Methanol vs. IPA**	**Ethanol vs. IPA**
0	0.850	0.880	0.820
0.22	0.842	0.819	0.864
0.27	0.757	0.771	0.843
0.32	0.833	0.792	0.893

The SSIM values demonstrate that the emission patterns retain similarity across different refractive index environments, even as *d*/*a* increases. This indicates that despite

changes in the surrounding medium, the emission profile is preserved, confirming that the same resonance mode is being tracked. Using SSIM in this analysis provides a quantitative assessment of mode consistency and supports the conclusion that the emission profile is largely unaffected by changes in the surrounding liquid. Furthermore, the SSIM values for *d*/*a* ratios from 0 to 0.32 show moderate to high similarity (ranging from 0.757 to 0.893), indicating that the emission profile remains consistent even with increasing the *d*/*a* ratio. The relatively stable SSIM values suggest that the core modal properties of the microdisk are not significantly impacted by subtle changes in the refractive index of the surrounding liquid environments.

[S1] Z. Wang, A. C. Bovik, H. R. Sheikh, and E. P. Simoncelli, "Image quality assessment: From error visibility to structural similarity," *IEEE Transactions on Image Processing*, vol. 13, no. 4, pp. 600–612, 2004, doi: 10.1109/TIP.2003.819861.

[S2] J. Peng *et al.*, "Implementation of the structural SIMilarity (SSIM) index as a quantitative evaluation tool for dose distribution error detection," *Medical Physics*, vol. 47, no. 4, pp. 1907–1919, 2020, doi: 10.1002/mp.14010.
